# Palliative care beyond traditional boundaries: A nationwide survey of bereavement support in the form of conversations and Palliative Care Registry utilization at Swedish stroke units

**DOI:** 10.1177/26323524261439943

**Published:** 2026-04-18

**Authors:** Sophie Mårtensson, Åsa Rejnö

**Affiliations:** 1 Department of Health Sciences, University of Skövde, Sweden; 2 Department of Health Sciences, University West, Trollhättan, Sweden; 3 Skaraborg Institute of Research and Development, Skövde, Sweden; 4 Department of Medicine, Skaraborg Hospital, Skövde, Sweden

**Keywords:** acute stroke, cross-sectional study, equality in care, quality care registry, quantitative descriptive design

## Abstract

**Background:**

Globally, stroke is the second leading cause of death. About 20% of patients with acute stroke die within 30 days of onset, yet fewer than 10% receive palliative care, and only a small fraction of these receive it in specialized palliative units. Rather, they are treated in stroke units, where both knowledge of palliative care and support for bereaved family members may be inconsistent and limited.

**Objectives:**

To describe the occurrence and design of bereavement support in the form of conversations offered to family members of patients who die from acute stroke in a stroke unit, as well as the registration in the Swedish Register of Palliative Care.

**Design:**

This study employed a quantitative descriptive design with a cross-sectional approach.

**Methods:**

A study-specific survey consisting of three main questions and several follow-up questions was used. All 72 stroke units in Sweden were contacted in person, by phone, or by email; 71 units ultimately participated.

**Results:**

Bereavement support in the form of conversations was provided to the family members of patients who had died from an acute stroke in 34 (48%) of the 71 stroke units. Among these, 21 units offered bereavement support to family members in all deaths, while 13 provided it when the need for support was identified. In total, 60 of the 71 stroke units (84%) register in the Swedish Register of Palliative Care, and of these, 25 units systematically use the registered data.

**Conclusion:**

Neither bereavement support in the form of conversations nor the systematic use of the palliative care registry are fully established part of care at stroke units. To ensure good and equitable end-of-life care, support for bereaved family members needs to be strengthened. Palliative registry data from stroke units represent an underutilized resource with the potential to improve both palliative care and bereavement support.

## Plain language summary

Exploring bereavement support and use of the Palliative Care Registry in Swedish stroke units: A nationwide survey

*Why was the study done?* Stroke is one of the leading causes of death worldwide. Many people who suffer a severe stroke die within a month, but only a few receive palliative care, specialized support for patients and their families at the end of life. Most are cared for in stroke units, where support for grieving family members can vary and may be limited.

*What did the researchers do?* This study looked at how stroke units in Sweden support families after a patient dies. Researchers contacted all 72 stroke units in the country, and 71 responded. Three main questions and follow-up questions were posed.

*What did the researchers find?* Less than half (48%) of the stroke units regularly offered conversations to support grieving families. Some units did this for every death, while others only offered support when they felt it was needed. Most stroke units (84%) also report data to the Swedish Register of Palliative Care, but only a few use this data to improve care.

*What do the findings mean?* This study shows that support for grieving families and the use of palliative care data are not yet standard practice in stroke units. Strengthening these areas could help ensure better and fairer care for patients and their families.

## Introduction

When individuals experience an acute and life-threatening stroke, it is crucial for healthcare professionals to address the need for palliative care.^1,2^ Generally, the needs of both dying patients and their family members can be addressed by healthcare professionals with basic knowledge of palliative care, referred to as general palliative care. Patients with more complex symptoms and needs should ideally be cared for by healthcare professionals with specialized knowledge in palliative care, known as specialized palliative care.^3^ However, Steigleder et al.^1^ showed that access to specialized palliative care is often neglected for individuals who are afflicted by stroke. Most patients who die during the acute phase of a stroke are not treated in specialized palliative care units, but rather in stroke units, where knowledge of palliative care may be inconsistent and limited. A systematic review found that 20% of patients with acute stroke die within 30 days, yet fewer than 10% receive palliative care.^4^ This can impact both the care provided to the patient and the support offered to their family members. The way healthcare professionals support families during a life-threatening stroke and after the patient’s death plays a significant role in helping them process the event.^4^ It also potentially reduces the risk of complicated and prolonged grief.^5^

Globally, stroke is the second leading cause of death^6,7^ while in Sweden, it ranks as third,^8^ and the incidence continues to rise worldwide.^6^ However, in several high-income countries, including Sweden, the incidence of stroke has been decreasing by approximately 1% annually since the 1970s, with a 1%–1.5% decrease over the past three decades.^9^ This is primarily attributed to improved control of risk factors.^10^ Riksstroke, the Swedish national quality registry for stroke care, collects nationwide data on the prevention, acute treatment, rehabilitation, and long-term outcomes of patients with stroke. Data from the registry show that stroke units are well established in Sweden. In 2024, 94% of the approximately 20,000 patients diagnosed with stroke (mean age 75 years) received such care.^11^

Evidence on early mortality (⩽7 days) after acute stroke in unselected populations, including both ischemic and hemorrhagic stroke, remains limited. Most available data concern ischemic stroke and indicate a decline in in-hospital mortality, from 6.98% in 2000 and 5.2% in 2010 to 3.1% in 2017, based on the Nationwide Inpatient Sample.^12,13^ A 7-day mortality rate of 2% has been reported using data from the Austrian Stroke Unit Registry.^14^ However, because admission to stroke units at that time was largely restricted to patients expected to benefit from acute treatment, early mortality was likely underestimated compared with unselected populations. Mortality following intracerebral hemorrhage is substantially higher, with a 1-month case fatality rate of 35.5% reported in a systematic review.^15^ Taken together, these findings indicate that early mortality after stroke remains a significant clinical concern, particularly when all stroke types and unselected populations are considered. This underscores the importance of palliative care competence within acute stroke units.

Initially, the prognosis after a massive acute stroke is uncertain, and the patient’s condition can fluctuate rapidly.^1^ This may create uncertainty regarding whether the patient requires general palliative care or if the patient will survive.^16–18^ The initial uncertainty on prognosis and the recognition that patients with neurologic diseases have unique needs have shed light on stroke as a disease with palliative care needs.^2,4,16^ Loss of consciousness, a well-established predictor of death following stroke,^19^ remains a significant factor today.^20,21^ In addition, patients with life-threatening stroke often experience cognitive impairment and reduced communication ability,^22^ limiting their capacity to express care preferences or participate in decision-making.^18^ Consequently, family members frequently act as proxies, representing the patient’s prior wishes regarding care and end-of-life decisions.^3,23,24^ Dedicated palliative care units might not be the best choice to meet these needs. International stroke clinical practice policy and guidelines emphasize the urgent need to integrate palliative care principles into acute stroke management.^2,24,25^ These principles emphasize relief of distressing symptoms, support for communication and shared decision-making, attention to psychological and social needs, and care that aligns with the patient’s values and goals.^26^ Barriers for this include healthcare professionals’ lack of knowledge about what palliative care actually is, and their discomfort in discussing it with the patient and their family members.^1,24^ Furthermore, family members of those who died from a stroke experience reduced quality of life as a result of the emotional and practical strain involved.^27^ This finding is consistent with work emphasizing that lack of information from healthcare professionals during a life-threatening stroke and after death may lead to prolonged grief.^28^

The palliative care process involves the early identification, assessment, and treatment of physical, psychological, social, and existential needs. These needs are addressed independently of patient characteristics such as diagnosis, age, sex, disability, ethnicity, religion, or other demographic or health-related factors. ‘Early’, in this context, means prompt recognition from admission or first clinical contact, while ‘treatment’ refers to the management of identified symptoms and needs. Family members ought to be given time and support in a situation where the transition between life and death, because of an acute stroke, can be sudden and overwhelming.^1,29,30^ In addition, the palliative care process includes offering bereavement support to family members.^1,3,26,31^ According to Swedish guidelines, bereavement support includes offering family members an organized support conversation (hereafter referred to as bereavement support in the form of conversation), with healthcare professionals either at the time of death or at a time thereafter.^32,33^ During the bereavement conversation, family members have the opportunity to be listened to and to ask questions about the situation. Healthcare professionals can, in turn, assess the need for additional support, identify potential risk for complicated grief, and, when appropriate, make referrals for continued support.^3^ The Swedish Register of Palliative Care is a national quality registry that collects data on end-of-life care across healthcare settings in Sweden. Its aim is to monitor and improve the quality of palliative care, regardless of diagnosis, age, sex, geographical location, and level of care. This is achieved by providing systematic information on symptom management, communication, care planning, and other key aspects of care during the last days of life. Healthcare professionals document patients’ care during the last week of life in the register, based on key qualities of good end-of-life care. The register also includes a post-bereavement survey for family members, collecting relatives’ assessments of the quality of end-of-life care during the patient’s last days, including whether bereavement support through conversation was offered.

It has long been recognized that specialized palliative care benefits family members of patients with various diagnoses. Compared with general palliative care, families report better fulfillment of emotional and spiritual needs^35^ and enhanced self-efficacy.^34,35^ These principles can be useful for stroke units to identify ways to support family members, including offering elements consistent with high-quality palliative care, such as bereavement support,^3^ particularly for children, adolescents, and individuals at risk of prolonged grief.^5^ To our knowledge, no study currently reports on bereavement support for family members after a patient has died from an acute stroke, nor are there studies on the registration and utilization of palliative care quality registers within this context. Therefore, this study aims to describe the occurrence and design of bereavement support in the form of conversations offered to family members of patients who die from acute stroke in a stroke unit. In addition, it also aims to describe the registration in the Swedish palliative care register.

## Method

The aim of the study was explored using a quantitative descriptive design with a cross-sectional approach^36^ utilizing a study-specific survey. The study included all 72 stroke units in Sweden, identified through Riksstroke 2023. The reporting of the study follows the STROBE statement checklist for cross-sectional studies^37^ (Supplemental File 1).

### Survey

Since there were no validated surveys suitable for exploring the research question, a survey was developed by the authors with two focus areas: bereavement support in the form of conversations with family members and reporting to and utilization of the national quality registry Swedish Register of Palliative Care (Supplemental File 2). In this study, the term ‘family member’ is defined as individuals close to the patient, regardless of whether the relationship is formalized by kinship. To assess face validity, the survey questions were pilot tested with two healthcare professionals specializing in stroke care, both with expert knowledge in the field. Their review did not indicate a need for any revisions. The survey took approximately 10–15 min to complete. The pilot testing resulted in minor clarifications of wording. The final survey included three main questions: (i) *Do you have a structured process in your stroke unit for following up family members of patients who have died from a stroke? This is often referred to as bereavement support in the form of conversations*. (ii) *Do you register in the Palliative Care Registry?* (iii) *Do you use your own data from the Palliative Care Registry?* If a main question was answered with ‘yes’, open-ended follow-up questions were asked (Supplemental File 1).

### Data collection

Healthcare professionals at the 72 stroke units in Sweden were approached between November 2023 and March 2024. Units were included if they met the criteria for being a stroke unit during the study period. A stroke unit is defined as a specialized, geographically defined hospital unit dedicated to the management of stroke patients^38^ where work in multidisciplinary teams is emphasized.^24,25,39^ Units that did not meet these criteria, that is, were not stroke units, were not included. Data collection was conducted by both authors, with the stroke units divided between them. Personal contact or phone calls were used for individuals at units within the last author’s extended network, with the survey answered orally. All other units were contacted by email and completed the survey in writing. If the initially contacted individual was unable to respond, they referred the authors to an appropriate colleague. Non-responders to the initial email received up to three reminders before a phone follow-up. In total, responses were obtained from 71 stroke units.

### Data analysis

Statistical analyses were performed using Excel for Windows Office 2013. The values for the survey variables are data on a nominal scale. Complete data was available for the three main questions, while responses to the follow-up questions were occasionally missing. Data were analyzed using descriptive statistics, including frequencies and percentages, presented in the results as bar graphs. The responses to the open-ended questions were generally brief, typically only a few words or a single sentence. As a result, an in-depth analysis was not feasible, and the answers are instead compiled and presented in textual form.

## Result

Results on bereavement support are presented first, followed by those on palliative care registration and utilization. Each section opens with the quantitative findings and continues with the free-text responses. To illustrate key points, quotes are provided; ‘RU’ and a number are used to denote the responding unit.

### Bereavement support

**Figure 1.**
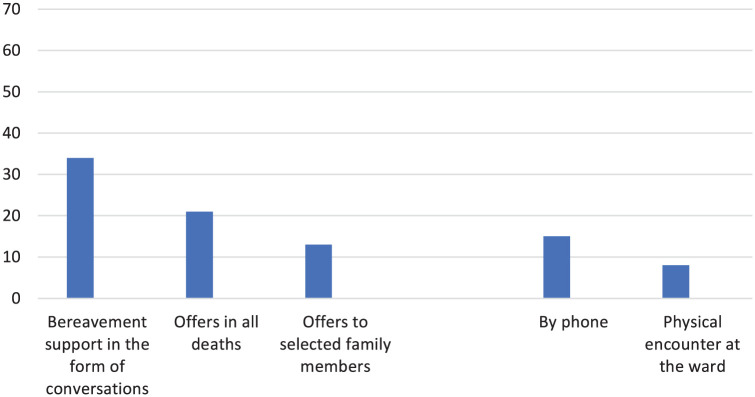
Bereavement support offered at the participating stroke units.

Bereavement support in the form of conversations was provided to the family members of patients who had died from an acute stroke in 34 (48%) of the 71 stroke units. Among these, 21 units stated that they offer bereavement support to family members in all deaths, while 13 provided it only where the need for support was subjectively identified by one (or possibly more) of the healthcare professionals at the unit (Figure 1). Free-text responses revealed that where a need was identified through individual subjective assessments, some units facilitated contact with a hospital social worker or religious representative. Moreover, responses indicated that working proactively and providing family members with continuous information during the palliative care, was beneficial and lessened the need for bereavement support. Hence bereavement support in the form of conversations were deemed to not be needed.

Family members receive information before and during the palliative care, so usually they don’t have too many questions. But, of course, questions may arise over time. (RU57)

It also came forth that several of the stroke units required family members to initiate contact themselves. It was also acknowledged that bereavement support was occasionally overlooked, and the participants identified opportunities for improvement in this area. Of the 34 units offering bereavement support in the form of conversations, 15 stated that these were routinely conducted by phone, with the option of meeting at the unit if preferred by the family members. A further eight units provided the conversations as in-person encounters on the ward. Who held the conversation could vary, but most often it was held by physicians (*n* = 5), medical social workers (*n* = 4), or registered nurses (*n* = 2). Some units (*n* = 2) had the routine that the nurse who cared for the patient at the time of death held the bereavement support conversation. Some responses indicated that newly graduated registered nurses could be hesitant to hold the conversations, which sometimes led to the process failing. Other units had a specially designated person responsible for all bereavement support conversations, often with deepened knowledge and interest in palliative care. Several of the units stated that the conversations were offered by the physician to family members, after saying goodbye to the deceased, but before they left the unit. Some responses indicated that this usually occurred when the stroke was more extensive or when a goals-of-care conversation marking the transition from disease-directed treatment to end-of-life care had not been conducted with family members.

Of the 37 stroke units that did not offer bereavement support, several free-text responses stated that the units intended to implement it as part of planned improvement projects. In addition, several units mentioned that if family members contacted them, they offered the possibility of such a conversation.

Bereavement support in the form of conversations is offered sporadically, primarily upon request, and there is clear potential for improvement. (RU27)

The free-text responses revealed that many units, regardless of providing bereavement support or not, routinely provided a booklet, ‘*What do I do now? A small booklet to read at the time of death*’, to bereaved family members when they leave the unit after a death. This booklet, supplied by the Swedish Funeral Directors’ Trade Association, explains what happens after death from a practical perspective. It covers topics such as what happens to the deceased, what needs to be done when someone dies, and what to consider before meeting with the funeral home. This was considered as a support to the bereaved family members.

### The Swedish Register of Palliative Care and utilization of its data

**Figure 2.**
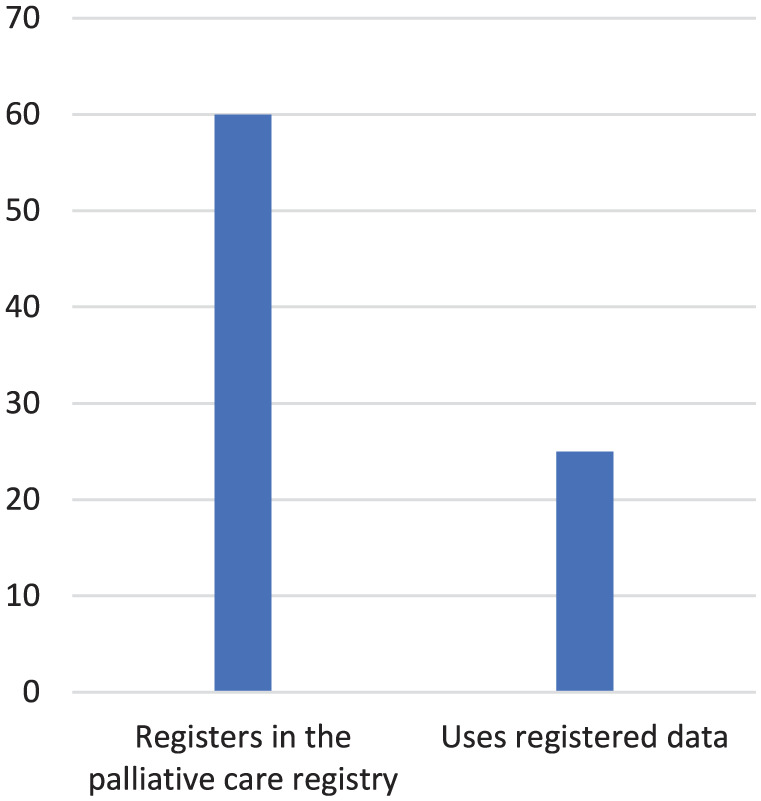
Participating stroke units registering in the palliative care registry and use of registered data.

In total, 60 stroke units of the 71 (84%) units registered in the Swedish Register of Palliative Care (Figure 2). The free-text responses revealed that several of the units had previously reported to the registry but were no longer doing so. This could be due to facts like that the person responsible for registration had resigned. Some units intended to stop registering, and others were examining the possibility of starting or were going to start. The Swedish Register of Palliative Care’s importance for stroke patients was doubted. Some stroke units had an ongoing discussion about whether it is reasonable to work with the Swedish Register of Palliative Care, since the stroke care registry Riksstroke was deemed to be of more importance for them.

Of the 60 units reporting data to the palliative care register, 25 stroke units systematically used the registered data (Figure 2). The free-text responses revealed that several units regularly reviewed the data during workplace meetings. Additionally, responses indicated that quality improvement projects had been initiated in response to poor results in certain aspects of the registered data. Two examples of such projects were provided: one involved pain assessment, and the other involved developing a schedule for oral health assessments.

We review the data and present it at staff meetings. Actions have been taken in response to poor data quality, for example by adding a column for pain assessment on the turning schedule. (RU25)

It was also reported that medically related variables, such as pain assessments, were given more attention than other registered variables. Further, responses revealed that systematic use of data was lacking in some units. Some had not found effective ways to work with the data, while others were unsure of how to use data. It was also noted that key individuals responsible for registration had resigned, resulting in data no longer being utilized.

## Discussion

The results point to palliative care as an underprioritized aspect of stroke unit care, with the potential to improve end-of-life care for patients with severe acute stroke. As seen in the results, knowledge of palliative care, its foundational principles, and process appears to be insufficient in acute stroke unit care. Similar results have also been reported from the UK context.^40^ One example of insufficient knowledge identified in the present study is the misconception regarding bereavement support in the form of conversation, as revealed by the survey. It is emphasized that the transition of care conversation and the provision of information during the care preceding death would negate the need for bereavement support in the form of conversations. However, this view contradicts current Swedish guidelines, which explicitly emphasize bereavement conversation as an important support for family members.^3^ It is also crucial to recognize that the need for support cannot be accurately assessed solely based on healthcare professionals’ perceptions of the amount of information delivered during patients’ end of life. Provision of information while the patient is still alive and bereavement support in the form of conversation after death are distinctly different, each serving unique purposes. The results also show that healthcare professionals tend to believe they can accurately assess family members’ need for support after a death, including bereavement support, without directly asking the family members themselves. It is emphasized that bereavement support in the form of conversations may provide family members with opportunities to process what has happened and find answers.^41^ This has the potential to avoid becoming trapped in prolonged grief.

The processes described by the stroke units do not always adequately support the provision of bereavement support in the form of conversations. While the processes might seem good in theory, for example, assigning the nurse responsible for the patient’s care at the time of death to provide bereavement support based on their established relationship with the family, these processes often prove challenging to implement in practice. This difficulty arises, for example, in cases where less experienced nurses are the ones who have cared for the patient, as they can feel that it is too difficult to have these conversations. Scheduling can also make it difficult to find a meeting time when the nurse responsible is on duty and the family members are available. In addition, stroke units that designate a specific individual to handle all bereavement support report fewer challenges, although issues still exist, such as when that individual is temporarily absent or resigns. This shows that simply having established routines in place is not sufficient. Building redundancy into processes is essential to prevent failures when they are under strain.^42^

For both bereavement support in the form of conversations and registration in the palliative care registry, the presence of individual enthusiasm was identified as a key driving force, a so-called champion. Such individuals have been shown to have a crucial role in sustaining healthcare interventions.^43^ The results indicate that when such enthusiastic individuals leave a unit, interest in these activities often diminishes, and established processes may deteriorate. In some cases, this has led to the complete discontinuation of both bereavement support in the form of conversations and data registration and use, even in units that had previously demonstrated good compliance. While individual enthusiasm plays a crucial role in initiating and mobilizing engagement among staff, it should not be the sole foundation for sustaining practices over time. To ensure long-term continuity and reliability, enthusiasm must eventually be transformed into established processes that are clearly integrated into the organizational structure. This reduces the risk of becoming dependent on specific individuals and promotes a sustainable, systematized approach to quality improvement and palliative care principles.

The results show that a majority (58%) of Swedish stroke units do not actively use the data they register in the Swedish Register of Palliative Care. Registration in quality care registries is a valuable tool for monitoring the quality of care provided.^44^ However, registering alone is not sufficient; there must also be a systematic effort to utilize data with the aim to maintain and improve quality when deficiencies are identified. Addressing such deficiencies should be an integral part of utilizing the full potential of quality care registers. This suggests that opportunities to identify and act upon shortcomings in end-of-life care are being missed at Swedish stroke units, thereby limiting the ability to implement meaningful improvements.

There are several concerns associated with collecting data that are not subsequently utilized. One major issue is the inefficient use of healthcare professionals’ time, particularly concerning in today’s healthcare environment, where a shortage in staff is a global and growing challenge.^45^ Time spent entering data that does not inform practice could instead be directed toward more meaningful and patient-centered care activities. Furthermore, there is an ethical dimension to consider: collecting data that is used only to a limited extent raises questions about transparency, accountability, and resource allocation.

For quality registers to be truly effective, they must have high coverage and consistent participation across healthcare units. When registration is incomplete or inconsistently performed, the reliability and overall value of the data are reduced.^46^ It is, therefore, essential that all stroke units contribute to the legitimacy and utility of the registry by not only participating in data collection but also actively engaging with the results to guide improvements in care.

In the context of stroke care in Sweden, a complicating factor is the existence of two separate quality registries: Riksstroke, the national quality register specifically dedicated to stroke care, and the Swedish Register of Palliative Care, which is designed to capture data on deaths across all diagnoses and specialties. All stroke units are expected to register in Riksstroke, while the palliative register requires registration of all deaths, regardless of cause, to be reported by all healthcare providers. As a result, stroke units are expected to contribute to both systems. In 2024, the coverage rate for Riksstroke was 87%,^11^ whereas the coverage rate for the Swedish Register of Palliative Care remains considerably lower, 60% for 2024.^47^ Notably, the present study found that 83% of responding stroke units report registering in the palliative care register. One plausible explanation for this high level of participation is the established process of registration within Riksstroke, which may foster a general culture of registration and quality monitoring. While it may be seen as less problematic that a smaller proportion of stroke units actively use their own data for quality improvement, the act of registering itself holds inherent value. Simply participating in the palliative care registry can help draw attention to palliative needs in stroke care and foster a greater focus on this aspect of treatment. Even if the collected data are not systematically analyzed at the local level, registration helps ensure that stroke is recognized as a condition associated with significant palliative care needs. This is critical, as stroke remains one of the leading causes of death. Importantly, the results also indicate that organizations actively using their data are better positioned to identify shortcomings in care and to design interventions aimed at improving the quality of care. This underscores the potential of quality registers, not only as monitoring tools but also as strategic instruments for driving evidence-based improvements in patient outcomes. Future studies focusing on family members and healthcare professionals’ experiences of bereavement support in the form of conversations at stroke units would be welcome.

### Strength and limitations

This study has some limitations, primarily related to survey design and the varied ways of distribution. Stroke units contacted in person or by phone provided more detailed responses, whereas those reached only via email tended to give shorter answers, limiting opportunities for clarification. Nevertheless, the multiple contact methods contributed to a high response rate, with only one unit not replying, which can be considered a strength. Responses from all participating units were obtained for the three main questions; however, not all units answered all follow-up questions. Consequently, findings from the follow-up questions should be interpreted with caution, and their generalizability at the national level may be limited. Because of the national perspective, the results may not be generalized to the global perspective of stroke unit care. However, the results may be relevant to countries with similar healthcare organizations and comparable development of stroke units and palliative care services.

## Conclusion

Palliative care at stroke units, when patients die during the acute phase of a stroke, falls beyond traditional boundaries of palliative care and regular stroke unit care. Bereavement support in the form of conversations and the systematic use of the palliative care registry are not yet fully established components of end-of-life care at stroke units. Strengthening bereavement support in the form of conversations for bereaved family members is essential to ensure the key qualities of good end-of-life care and equitable palliative care. In parallel, systematic quality improvement efforts based on data from the Swedish Palliative Care Register need further development. The registry data represent an underutilized resource with the potential to improve both palliative care at the individual stroke unit and bereavement support for family members.

## Supplemental Material

sj-docx-1-pcr-10.1177_26323524261439943 – Supplemental material for Palliative care beyond traditional boundaries: A nationwide survey of bereavement support in the form of conversations and Palliative Care Registry utilization at Swedish stroke units Supplemental material, sj-docx-1-pcr-10.1177_26323524261439943 for Palliative care beyond traditional boundaries: A nationwide survey of bereavement support in the form of conversations and Palliative Care Registry utilization at Swedish stroke units by Sophie Mårtensson and Åsa Rejnö in Palliative Care and Social Practice

sj-docx-2-pcr-10.1177_26323524261439943 – Supplemental material for Palliative care beyond traditional boundaries: A nationwide survey of bereavement support in the form of conversations and Palliative Care Registry utilization at Swedish stroke units Supplemental material, sj-docx-2-pcr-10.1177_26323524261439943 for Palliative care beyond traditional boundaries: A nationwide survey of bereavement support in the form of conversations and Palliative Care Registry utilization at Swedish stroke units by Sophie Mårtensson and Åsa Rejnö in Palliative Care and Social Practice
