# Supplementary material for: Palliative care beyond traditional boundaries: A nationwide survey of bereavement support in the form of conversations and Palliative Care Registry utilization at Swedish stroke units
Source: Palliat Care Soc Pract. 2026 Apr 18;20:26323524261439943. doi: 10.1177/26323524261439943 (PMC13100386; doi:10.1177/26323524261439943)
Supplement: sj-docx-2-pcr-10.1177_26323524261439943 – Supplemental material for Palliative care beyond traditional boundaries: A nationwide survey of bereavement support in the form of conversations and Palliative Care Registry utilization at Swedish stroke units [file sj-docx-2-pcr-10.1177_26323524261439943.docx]

Supplemental file 1

**Survey Questionnaire**

1. *Do you have a structured process in your stroke unit for following up family members of patients who have died from acute stroke? This is often referred to as bereavement support in the form of conversations.*
2. *Do you register in the Palliative Care Registry?*
3. *Do you use your own data from the Palliative Care Registry?*

If a main question was answered with 'yes', open-ended follow-up questions were asked;

1. *Is bereavement support in the form of conversations offered at all deaths?*
2. *How is the bereavement support in the form of conversations offered?*
3. *By whom is the bereavement support in the form of conversations offered?*
4. *How do you use your own data from the Palliative Care Registry?*
